# Status of Common Water-Soluble Vitamins in Medical Students: A Cross-Sectional Analysis with Implications for Targeted Nutritional Screening Programs

**DOI:** 10.3390/nu17172862

**Published:** 2025-09-04

**Authors:** Adnan Agha, Javed Yasin, Charu Sharma, Juma Alkaabi

**Affiliations:** 1 Department of Internal Medicine, College of Medicine and Health Sciences, United Arab Emirates University, Al Ain 15551, United Arab Emirates; 2 Department of Genetics and Genomics, College of Medicine and Health Sciences, United Arab Emirates University, Al Ain 15551, United Arab Emirates

**Keywords:** water-soluble vitamins, medical students, vitamin C, vitamin B12, nutritional screening, young adults, cross-sectional study, micronutrient deficiency, academic stress

## Abstract

Background/Objectives: Water-soluble vitamins are essential micronutrients requiring regular dietary replenishment due to minimal body storage capacity. Medical students.; despite their health knowledge, may be at risk for subclinical deficiencies due to academic stress and life-style factors. This study assessed water-soluble vitamin status to evaluate screening needs in this educated population. Methods: A cross-sectional study was conducted among 91 healthy medical students (age 18–23 years) at UAE University from September 2023 to January 2024. Serum levels of folate (B9), cobalamin (B12), and ascorbic acid (Vitamin C) were measured using validated high-performance chemiluminescent immunoassays. Demographic, anthropometric, dietary, and lifestyle data were collected via structured questionnaires. Statistical analyses included multivariate logistic regres-sion, correlation analyses, and receiver operating characteristic (ROC) curves. Results: Among the participants (70.3% female; mean age 19.8 ± 1.4 years; BMI 23.2 ± 2.9 kg/m^2^), vita-min C showed the highest prevalence of suboptimal levels at 7.7% (7/91 participants), comprising 2.2% with deficiency (<28 µmol/L, *n* = 2) and 5.5% with insufficiency (28–40 µmol/L, *n* = 5). Mean vitamin C was 56.7 ± 14.8 µmol/L. Vitamin B12 insufficiency (200–300 pg/mL) affected 9.0% (8/89) of students, with a mean of 485.3 ± 165.0 pg/mL. A non-significant trend toward higher insufficiency rates was observed among female students. No deficiencies were observed for folate (mean 14.1 ± 4.9 ng/mL). Multivariate analysis identified low fruit/vegetable intake (OR 4.8; 95% CI: 1.3–17.6; *p* = 0.018); high stress scores (OR 3.2; 95% CI: 1.1–9.4; p = 0.033); and female gender (OR 2.9; 95% CI: 0.9–9.1; *p* = 0.071) as predictors of suboptimal vitamin C. Vitamin C levels correlated positively with dietary quality (r = 0.412; *p* < 0.001) and negatively with stress scores (r = −0.241; *p* = 0.031). Despite being a health-educated population, nearly 10% of medical students demonstrated suboptimal water-soluble vitamin levels, particularly vitamins C and B12. Conclusions: These findings support implementing targeted screening programs focusing on high-risk groups, including students with poor dietary habits, high stress levels, or specific gender-based risks.

## 1. Introduction

### 1.1. Water-Soluble Vitamins: Biochemical Roles and Clinical Significance

Water-soluble vitamins comprise eight B-complex vitamins and vitamin C, distinguished from fat-soluble vitamins by their hydrophilic properties, limited tissue storage capacity, and rapid urinary excretion [1]. These characteristics necessitate regular dietary intake to maintain optimal physiological function and prevent deficiency states. The B-complex vitamins function primarily as enzymatic cofactors in critical metabolic pathways, including energy metabolism, DNA synthesis and repair, neurotransmitter production, and hematopoiesis [2,3].

Cobalamin (vitamin B12) and folate (vitamin B9) work synergistically in one-carbon metabolism, facilitating methylation reactions essential for DNA synthesis, epigenetic regulation, and homocysteine metabolism [4,5]. B12 deficiency can cause megaloblastic anemia, peripheral neuropathy, and cognitive impairment, with subclinical deficiency (200–300 pg/mL) associated with elevated homocysteine and methylmalonic acid levels [6,7]. Folate deficiency, beyond its role in neural tube defects, impacts cellular division, immune function, and cardiovascular health through homocysteine accumulation [8].

Vitamin C (ascorbic acid) functions as a potent water-soluble antioxidant and enzymatic cofactor in collagen synthesis, carnitine biosynthesis, and catecholamine production [9]. It modulates immune function through support of epithelial barrier function, enhancement of phagocytic activity, and promotion of lymphocyte proliferation [10]. Subclinical deficiency, while not causing overt scurvy, is associated with fatigue, impaired wound healing, increased infection susceptibility, and potentially compromised stress response [11]. We selected vitamins B12, B9, and C for this study based on their synergistic roles in pathways particularly relevant to student populations: one-carbon metabolism essential for cognition (B12 and folate), antioxidant defense against stress-induced oxidative damage (vitamin C), and their well-established, reliable biochemical assays.

### 1.2. Nutritional Vulnerabilities and Global Prevalence

University students represent a paradoxical population, often exhibiting poor dietary practices despite theoretical health knowledge. Medical students face additional stressors, with demanding curricula requiring 60–80 h of weekly study and 25–75% experiencing significant psychological distress, creating a perfect storm for nutritional compromise [12,13]. A review of 35 studies found 20–58% of university students lacked adequate micronutrient intake [14]. Medical students, with 80-h weeks, face added stressors that increase nutritional risk [15,16]. They also report higher psychological distress than peers, affecting 25–75% [17].

Globally, vitamin C deficiency prevalence ranges from 5–23% in developed countries, with possibly higher rates in smokers and low-income populations [18,19]. B12 deficiency affects an estimated 2.5–26% of the general population, with young adults showing particular vulnerability [20,21]. Despite Gulf prosperity, Middle Eastern populations remain vulnerable to micronutrient deficiencies due to regional dietary habits, limited diversity, restrictive eating, and a shift toward energy-dense, nutrient-poor foods [22].

### 1.3. Study Rationale and Objectives

As both a vulnerable group and future healthcare providers, medical students face nutritional risks with significant public health implications. Data on water-soluble vitamin status in Middle Eastern populations remain limited with one study from Saudi Arabia reporting vitamin B12 deficiency in 24.9% of female university students [23]. This study aims to address this gap.

#### Primary Objective

The main objective of this study was to determine the prevalence of water-soluble vitamin deficiencies among medical students and identify key predictors of suboptimal status.

## 2. Materials and Methods

### 2.1. Study Design and Ethical Considerations

This cross-sectional observational study was conducted at the College of Medicine and Health Sciences, United Arab Emirates University, Al Ain, between September 2023 and January 2024. The study protocol received approval from the UAE University Research Ethics Committee (Reference: ERS_2023_9456) and was conducted in accordance with the Declaration of Helsinki principles and STROBE guidelines for observational studies as well TRIPOD statement for risk factor prediction [24]. All participants provided written informed consent.

The study’s secondary objectives were to examine relationships between dietary patterns and biochemical vitamin status, evaluate gender-specific patterns in vitamin deficiency, and provide evidence-based recommendations for targeted screening programs.

### 2.2. Participant Recruitment and Selection

#### 2.2.1. Sampling Strategy

A convenience sampling method was utilized for participant recruitment. An open invitation was extended to all 652 enrolled medical students across both preclinical and clinical years of the MD program via email reminders and display posters inside the college building. The final study sample was composed of students who responded to the invitation, met the eligibility criteria, and provided informed consent to participate.

#### 2.2.2. Participant Selection

A convenience sampling method was used, with an open invitation extended to all 652 enrolled medical students. Participants were eligible if they were aged 18 to 25 years and had not taken vitamin supplements for at least three months. Exclusion criteria are summarized in Table 1.

### 2.3. Sample Size Calculation

The required sample size was determined a priori using Cochran’s formula. An expected prevalence (P) of 15% was estimated based on previous studies on similar populations [19,20]. With a 95% confidence level (Z-score of 1.96) and a desired precision (d) of ±7.5%, the calculation yielded: *n* = (1.96^2^ × 0.15 × 0.85)/0.075^2^ = 87 participants. Applying the finite population correction for 652 enrolled students: n’ = *n*/(1 + (*n* − 1)/*n*) = 87/(1 + 86/652) = 77 participants. To accommodate for potential dropout, this number was inflated by approximately 10%, establishing a final recruitment target of 85 students. Our final analytical sample of 91 participants exceeded this requirement.

### 2.4. Data Collection Procedures

#### 2.4.1. Questionnaire Administration

A validated, structured questionnaire, developed by the research team and pilot-tested for clarity, was administered by trained research assistants to collect comprehensive data from each participant. The questionnaire was divided into four main sections:

##### Demographic and Socioeconomic Information

This section gathered data on participants’ age, gender, nationality, and current academic year. To provide socioeconomic context, information was also collected on parental education and occupation, household income category, and the student’s residential status, distinguishing between those living on or off-campus.

##### Relevant Medical History

The medical history portion of the questionnaire was used to document any past medical conditions, a list of current medications and supplements, and any known family history of nutritional deficiencies. Participants were also asked to report any recent illnesses or hospitalizations to account for acute health events that could influence vitamin status.

##### Lifestyle Assessment

A lifestyle assessment was conducted to evaluate key behavioral factors. Physical activity was quantified using the International Physical Activity Questionnaire - Short Form (IPAQ-SF). Sleep quality was assessed using three components from the Pittsburgh Sleep Quality Index (PSQI): subjective sleep quality, sleep duration, and daytime dysfunction. The assessment also recorded academic load and leisure habits, including average weekly study hours and a breakdown of recreational versus academic screen time. Questions on smoking status were also included.

##### Dietary Assessment

To obtain a thorough understanding of nutritional intake, a detailed dietary assessment was performed. This included questions on meal frequency and regularity, two non-consecutive 24-h dietary recalls. The assessment also quantified specific dietary habits, such as daily servings of fruits and vegetables, the frequency of fast food consumption, and typical beverage preferences (water, soft drinks, coffee/tea). Lastly, information regarding cooking frequency and preferred food preparation methods was collected.

#### 2.4.2. Anthropometric Measurements

Standardized anthropometric assessments were performed by trained personnel following WHO protocols [25]:

- **Height:** Wall-mounted stadiometer (SECA 213, SECA GmbH & Co. KG, Hamburg, Germany), measured to nearest 0.1 cm
- **Weight:** Calibrated digital scale (TANITA BC-418, TANITA Corporation, Tokyo, Japan), measured to nearest 0.1 kg
- **BMI:** Calculated as weight (kg)/height^2^ (m^2^)
- **Waist circumference:** Non-stretchable tape at midpoint between lowest rib and iliac crest
- **Hip circumference:** Maximum circumference over buttocks
- **Waist-to-hip ratio:** Calculated for central adiposity assessment
- **Body composition:** Bioelectrical impedance analysis (TANITA BC-418)

### 2.5. Laboratory Analyses

#### 2.5.1. Pre-Analytical Sample Handling and Stability

To ensure the stability of vitamin C and other analytes, strict pre-analytical protocols were followed. All participants provided samples after a minimum 10-h overnight fast. Venous blood samples (12 mL) were collected between 07:00–10:00 h in serum separator tubes (8 mL) for vitamin analyses and EDTA tubes (4 mL) for backup. Samples were immediately protected from light and centrifuged at 3600 rpm for 10 min within 2 h of collection. Serum aliquots were then immediately stored at −80 °C until batch analysis to minimize inter-assay variation and prevent degradation of labile analytes, particularly vitamin C.

#### 2.5.2. Vitamin Assays

Serum levels of water-soluble vitamins were quantified using the following specific methods and assays:

**Cobalamin (Vitamin B12):** This was measured using the Elecsys^®^ Vitamin B12 II kit (Elecsys® Vitamin B12 II, Roche Diagnostics, Basel, Switzerland) on a Cobas e immunoassay analyzer. This assay is a competitive immunoassay employing electrochemiluminescence (ECLIA) technology. The assay has a measuring range of 30–2000 pg/mL. The inter-assay coefficient of variation (CV) ranges from 2.8–4.4%.

**Folate (Vitamin B9):** It was quantified using the Elecsys^®^ Folate III kit (Elecsys® Folate III, Roche Diagnostics, Basel, Switzerland) on a Cobas-E immunoassay analyzer. The method is a competitive binding assay based on ECLIA technology, utilizing a recombinant folate binding protein (FBP). The assay has a measuring range of 0.6–20 ng/mL with an inter-assay CV between 2.8–5.3%.

**Ascorbic Acid (Vitamin C):** This was measured using the Human Vitamin C (VC) ELISA Kit, Catalog No. EK710149, (Human Vitamin C (VC) ELISA Kit, Elabscience Biotechnology Inc., Houston, TX, USA). This assay is a competitive Enzyme-Linked Immunosorbent Assay with a detection range of 0.312–20 ng/mL and a sensitivity of less than 0.188 ng/mL. The reported precision for the kit shows an intra-assay CV of <10% and an inter-assay CV of <12%.

The following cutoffs were used:

**Cobalamin (vitamin B12):** Normal > 300 pg/mL; Insufficiency 200–300 pg/mL; Deficiency < 200 pg/mL

**Folate (vitamin B9):** Normal > 5 ng/mL; Deficiency ≤ 5 ng/mL

**Ascorbic Acid (vitamin C):** Normal > 40 µmol/L; Insufficiency 28–40 µmol/L; Deficiency < 28 µmol/L

### 2.6. Statistical Analysis

#### 2.6.1. Data Management

All data were entered initially in specifically designed proforma and copied electronically into Microsoft Excel. Discrepancies were resolved by consulting original records. Data cleaning included range checks, consistency verification, and outlier identification using the Tukey method.

#### 2.6.2. Statistical Methods

All data were analyzed using SPSS version 29.0 (IBM Corp., Armonk, NY, USA). Descriptive statistics were generated to summarize the data; continuous variables were presented as mean ± standard deviation (SD) or median (interquartile range, IQR) depending on their distribution (assessed using Shapiro–Wilk tests and Q–Q plots), while categorical variables were reported as frequencies and percentages.

For bivariate analyses, independent *t*-tests or Mann–Whitney U tests were used for continuous variables, and chi-square or Fisher’s exact tests for categorical variables. Correlations were examined using Pearson’s or Spearman’s coefficients as appropriate.

Multivariate analyses involved multiple linear regression for continuous vitamin levels and logistic regression for binary outcomes (deficiency/insufficiency). Models included pre-specified potential confounders (age, academic year, physical activity) regardless of their statistical significance. The Hosmer–Lemeshow test was used to assess goodness-of-fit for logistic regression models. Multicollinearity was assessed using variance inflation factor (VIF) with a cutoff of <5.

Receiver operating characteristic (ROC) curves were generated to evaluate the diagnostic accuracy of screening parameters, with 95% confidence intervals calculated for all areas under the curve (AUC). The Youden’s J statistic was used to identify the optimal cutoff points from the ROC curves. This method selects the risk score threshold that best balances the trade-off between sensitivity and specificity by maximizing their sum (J = Sensitivity + Specificity − 1). Given the exploratory nature of correlational analyses, we did not formally adjust for multiple comparisons. A two-tailed *p*-value of <0.05 was considered statistically significant for all primary analyses.

## 3. Results

### 3.1. Study Population Characteristics

**Figure 1:**
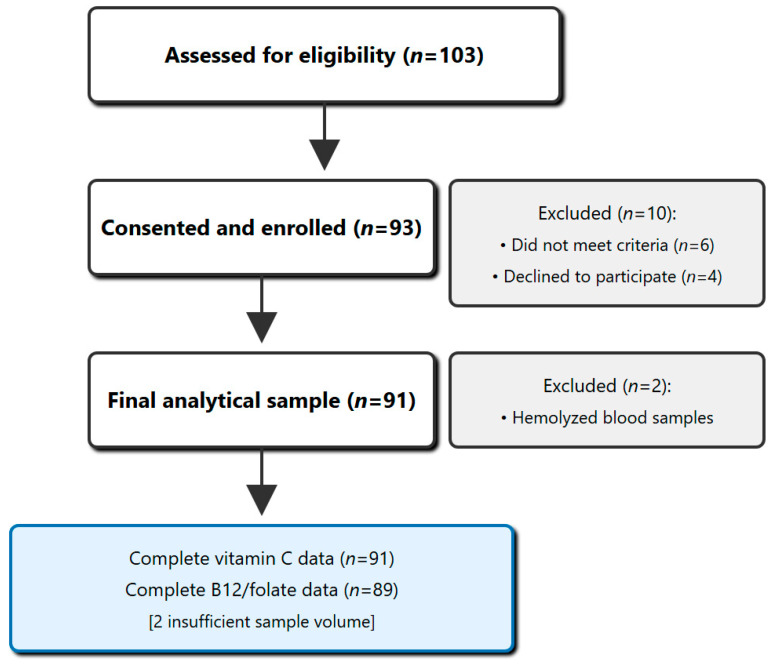
STROBE Flow diagram of the participants of this study. **Legend:** STROBE flow diagram showing participant recruitment, screening, and final analytical sample. Of 103 medical students initially screened, 91 provided complete data for analysis, representing an 88.3% response rate.

Of 103 students screened, 91 provided complete data (response rate: 88.3%), comprising 64 females (70.3%) and 27 males, with a mean age of 19.8 ± 1.4 years (Figure 1). Males had a significantly higher BMI than females (24.3 ± 3.1 vs. 22.7 ± 2.7 kg/m^2^, *p* = 0.018) and reported more physical activity (184 ± 92 vs. 124 ± 78 min/week, *p* = 0.002). Females reported significantly higher stress scores (7.3 ± 1.5 vs. 6.6 ± 1.7, *p* = 0.049); see Table 2 for baseline characteristics. All patients were UAE nationals; no significant associations were found between vitamin status and nationality, parental education, or household income (all *p* > 0.05).

The final analytical sample comprised 91 students with comprehensive vitamin assessments, see Figure 1 for the participants’ flow diagram.

### 3.2. Water-Soluble Vitamin Status

Analysis of vitamin levels reveals the deficiency or insufficiency of vitamin C and vitamin B12 in some participants (Table 3). Vitamin C showed the highest prevalence of suboptimal levels at 7.7% (7/91 participants), comprising 2.2% with deficiency (*n* = 2) and 5.5% with insufficiency (*n* = 5). The mean vitamin C concentration was 56.7 ± 14.8 µmol/L. Vitamin B12 insufficiency affected 9.0% of students (8/89), with a mean of 485.3 ± 165.0 pg/mL. No frank B12 deficiency was observed. All participants had normal folate levels (mean 14.1 ± 4.9 ng/mL).

### 3.3. Gender-Specific Patterns

While mean vitamin levels did not differ significantly between genders (B12: males 524.6 ± 178.3 vs. females 468.7 ± 157.2 pg/mL, *p* = 0.146), all seven cases of suboptimal vitamin C occurred exclusively in female participants (10.9% of females vs. 0% of males, *p* = 0.096). A non-significant trend toward a higher prevalence of B12 insufficiency was also observed among females (10.9% vs. 3.7%, *p* = 0.433). See Table 4.

### 3.4. Correlational Analyses

As shown in Table 5, vitamin C levels were strongly and positively correlated with fruit and vegetable intake (r = 0.412, *p* < 0.001) and negatively correlated with fast food frequency (r = −0.287, *p* < 0.01), stress score (r = −0.241, *p* < 0.05), and BMI (r = −0.198, *p* < 0.05). See Table 5.

### 3.5. Multivariate Predictors of Vitamin Status

**Figure 2:**
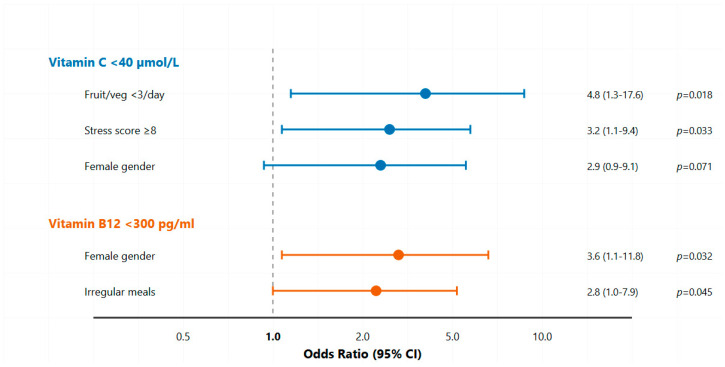
Multivariate Predictors of Suboptimal Vitamin Status. **Legend:** Cross-sectional study of water-soluble vitamin status in medical students at UAE University (September 2023–January 2024). The diagram illustrates participant flow from initial screening through final analysis following STROBE guidelines for observational studies. Abbreviations & Terms: *n*: Number of participants; UAE: United Arab Emirates; Vitamin C: Ascorbic Acid; Vitamin B12: Cobalamin; Folate: Vitamin B9; ROC: Receiver Operating Characteristic; BMI: Body Mass Index; SPSS: Statistical Package for Social Sciences.

Multivariate logistic regression identified distinct risk profiles (see Figure 2). Students consuming <3 servings of fruits/vegetables daily had 4.8-fold higher odds of suboptimal vitamin C (95% CI: 1.3–17.6, *p* = 0.018). High stress (score ≥ 8) increased odds 3.2-fold (95% CI: 1.1–9.4, *p* = 0.033). For B12 insufficiency, female gender (OR 3.6, 95% CI: 1.1–11.8, *p* = 0.032) and irregular meals (OR 2.8, 95% CI: 1.0–7.9, *p* = 0.045) were significant predictors. See Table 6 for more details.

### 3.6. Diagnostic Accuracy of Screening Parameters

**Figure 3:**
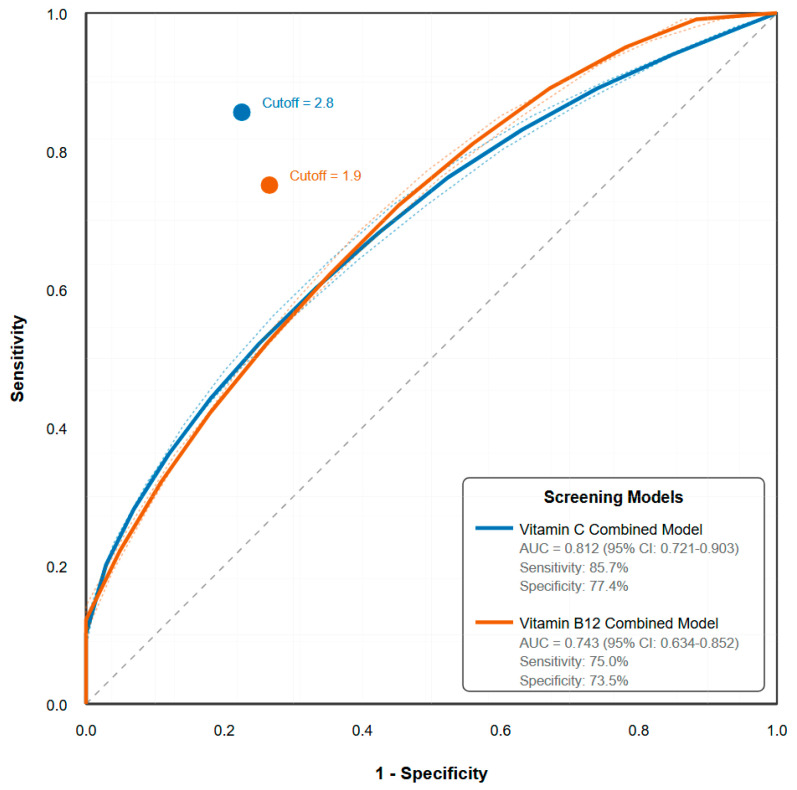
Receiver Operating Characteristic (ROC) curves for Vitamin C and Vitamin B12 Deficiency Prediction. The vitamin C model (blue line) combines dietary assessment (fruit/vegetable intake < 3 servings/day) and stress score (≥8/10), achieving an area under the curve (AUC) of 0.812. The vitamin B12 model (orange line) combines gender and meal pattern assessment, with an AUC of 0.743. Optimal cutoff points determined by Youden’s J statistic are marked with circles. The diagonal reference line represents no discriminatory ability (AUC = 0.5). Dashed lines represent 95% confidence intervals calculated using DeLong’s method. **Legend:** Forest plot of multivariate predictors for suboptimal vitamin status. The plot displays the adjusted odds ratios (squares) and 95% confidence intervals (horizontal lines) for predictors of suboptimal vitamin C (<40 µmol/L) and vitamin B12 (<300 pg/mL) from the logistic regression models presented in Table 5. The vertical line at an odds ratio of 1.0 indicates no effect; predictors with confidence intervals that do not cross this line are statistically significant (*p* < 0.05).

ROC analysis demonstrated the feasibility of risk-based screening (Figure 3). A combined risk score for vitamin C (diet and stress) achieved good discriminatory power with an AUC of 0.812 (95% CI: 0.721–0.903), yielding a sensitivity of 85.7% and specificity of 77.4%, with a negative predictive value of 98.1%, which may suggest its potential in ruling out vitamin C insufficiency in this population.

In contrast, screening parameters showed modest performance for B12 insufficiency, with the best AUC reaching only 0.68, which likely reflects B12′s complex absorption and metabolism, and suggests that screening may require more comprehensive assessment or testing strategies (Table 7).

### 3.7. Clinical and Academic Correlates

As shown in Table 8, students with any suboptimal vitamin level reported significantly higher fatigue scores (7.4 ± 1.5 vs. 5.2 ± 1.8, *p* < 0.001), more frequent upper respiratory infections (3.0 ± 1.4 vs. 1.8 ± 1.1 episodes/year, *p* = 0.003), and greater difficulty with concentration (75.0% vs. 35.0%, *p* = 0.009).

## 4. Discussion

### 4.1. Principal Findings and Clinical Significance

This assessment reveals that despite theoretical health knowledge, approximately 10% of medical students demonstrated suboptimal levels of critical vitamins. The 7.7% prevalence of suboptimal vitamin C and 9.0% of B12 insufficiency are concerning given the participants’ educational background and future roles as healthcare providers [24,26]. Our analysis of nationality and socioeconomic variables revealed no significant associations with vitamin status, suggesting that nutritional vulnerabilities persist even among relatively privileged populations. The complete absence of folate deficiencies suggests effective fortification programs or dietary patterns specific to the UAE context [27].

### 4.2. Vitamin C: Stress, Diet, and Academic Performance

The observed vitamin C insufficiency prevalence aligns with the lower range of global estimates (5-25%) but exceeds expectations for health-conscious individuals [28]. The strong correlation between vitamin C levels and fruit/vegetable intake (r = 0.412, *p* < 0.001) validates our dietary assessment methodology and highlights the impact of dietary habits, with low intake increasing the odds of suboptimal status by 4.8 times. The inverse relationship with stress scores (r = −0.241, *p* = 0.031) corroborates evidence that psychological stress increases vitamin C requirements through multiple mechanisms: enhanced oxidative stress, increased adrenal consumption for catecholamine synthesis, and potentially altered dietary behaviors during stressful periods [29]. This may create a vicious cycle where academic pressure depletes vitamin C, compromising stress resilience. The association with increased upper respiratory infections (3.0 vs. 1.8 episodes/year) suggests that even subclinical insufficiency may impact immune competence.

### 4.3. Vitamin B12: Gender Disparities and Subclinical Implications

The 9.0% prevalence of B12 insufficiency, while not reaching frank deficiency levels, warrants attention given the insidious nature of B12-related pathology. Levels between 200–300 pg/mL, though above traditional deficiency thresholds, are associated with elevated homocysteine and methylmalonic acid, markers of functional deficiency [30] The potential for subtle cognitive impairment, mood alterations, and long-term neurological consequences makes this finding particularly relevant for students requiring optimal cognitive function.

The gender disparity in B12 status, with females having 3.6 times the odds of insufficiency (OR 3.6, 95% CI: 1.1–11.8), aligns with broader epidemiological patterns. Several mechanisms may explain female vulnerability: lower meat consumption due to cultural or personal preferences, potential interactions with oral contraceptives affecting B12 metabolism, and the compound effects of iron deficiency on B12 absorption. The absence of serum iron measurements in our study is acknowledged as a limitation that prevents full exploration of this relationship.

Particularly striking was the finding that all seven cases of suboptimal vitamin C occurred exclusively in female participants. While the sample size was too small for definitive conclusions, this observation, combined with females’ higher stress scores (7.3 vs. 6.6, *p* = 0.049) and lower physical activity levels (124 vs. 184 min/week, *p* = 0.002), suggests a potential gender-specific vulnerability pattern that merits further investigation in larger studies.

### 4.4. Implications for a Targeted Screening Framework

Our findings suggest that a targeted screening approach may be more efficient than universal screening for water-soluble vitamins in university settings. The identification of specific, easily ascertainable risk factors, such as poor dietary habits (fruit/vegetable intake <3 servings/day), high stress levels (≥8/10), female gender, and irregular meal patterns, provides a foundation for developing risk stratification tools.

While our ROC analysis demonstrates promising discriminatory power, particularly for vitamin C (AUC 0.812), the low positive predictive value (28.6%) is an expected consequence of screening in a low-prevalence population and warrants careful consideration. Although the tool is effective at identifying a low-risk population that does not require further testing (NPV 98.1%), a positive indication should be considered an indication for confirmatory biochemical testing, not a diagnosis. Table 9 outlines a conceptual framework for a risk-stratified screening protocol.

While the proposed screening models demonstrate excellent discriminatory power, particularly for vitamin C (AUC 0.812), and a high negative predictive value that makes them effective for ruling out deficiency, the low positive predictive values (28.6% for vitamin C and 20.0% for B12) warrant careful consideration. This high false-positive rate is an expected consequence of screening in a low-prevalence population and highlights a critical trade-off. It signifies that while the tool is effective at identifying a low-risk population that does not require further testing, a positive screen should not be considered diagnostic.

### 4.5. Strengths and Methodological Rigor

This study’s strengths include its comprehensive assessment of three key vitamins using validated high-performance immunoassays, rigorous pre-analytical protocols ensuring sample stability, detailed collection of demographic, anthropometric, and lifestyle data enabling multivariate analyses, a high participation rate (88.3%) minimizing selection bias, and a theory-driven statistical approach incorporating pre-specified confounders in multivariate models.

### 4.6. Limitations and Future Directions

Several limitations merit consideration. The cross-sectional design precludes causal inferences about relationships between vitamin status and outcomes. The single-center setting and convenience sampling method in the UAE may limit generalizability to other Middle Eastern or global populations and may have introduced selection bias toward more health-conscious students. The absence of functional biomarkers (e.g., homocysteine, methylmalonic acid) and serum iron levels prevents a deeper metabolic assessment. The relatively small sample size, particularly for subgroup analyses, limits statistical power for detecting modest associations. Dietary assessment via questionnaire rather than detailed food records may underestimate true intake variability. The exploratory nature of our correlational analyses means these findings should be considered hypothesis-generating, as we did not formally adjust for multiple comparisons.

Future research should employ longitudinal, multi-center designs to assess temporal relationships between vitamin status and academic/health outcomes.

### 4.7. Public Health and Policy Implications

The identification of nutritional vulnerabilities in this educated group suggests the prevalence may be higher in the general young adult population. This highlights a gap between knowledge and practice and supports policy recommendations such as integrating nutrition education into curricula, increasing access to healthy foods on campus, implementing risk-stratified screening programs, and enhancing stress management initiatives.

## 5. Conclusions

This comprehensive analysis reveals that despite health education and awareness, approximately 10% of medical students demonstrate suboptimal water-soluble vitamin status, particularly vitamins C and B12. The strong associations with modifiable factors—dietary quality, stress levels, and meal patterns—suggest opportunities for targeted intervention. The complete absence of folate deficiency indicates that selective rather than universal deficiency patterns characterize this population. The study’s findings challenge assumptions about nutritional adequacy in educated populations and highlight that knowledge alone may be insufficient to ensure optimal nutritional status during demanding life phases. The development of risk-stratified screening protocols, targeting students with poor dietary habits or high stress levels, offers a practical approach to identifying and addressing subclinical deficiencies before clinical consequences emerge.

As future healthcare providers, medical students’ nutritional status impacts not only their personal health and academic performance but also their credibility as health advocates. Ensuring optimal nutrition in this population represents an investment in both individual wellbeing and the quality of future healthcare delivery. The integration of nutritional assessment and support into medical education infrastructure could serve as a model for broader young adult populations facing similar challenges in maintaining nutritional adequacy amidst academic and life stressors.

## Figures and Tables

**Table 1:** Participants’ Exclusion Criteria.

Category	Specific Exclusions
Medical Conditions	Diabetes, thyroid disorders, malabsorption syndromes, chronic kidney/liver disease
Medications	Proton pump inhibitors, metformin, isoniazid, phenytoin, phenobarbital
Physiological State	Pregnancy, lactation, acute illness within the preceding four weeks
Dietary Habits	Strict veganism, diagnosed eating disorders, regular alcohol consumption
Anthropometrics	Body Mass Index (BMI) < 18.5 or >30 kg/m^2^
Other	Were being treated for a previously diagnosed vitamin deficiency, current participation in another nutritional study

**Legend:** This table summarizes the criteria used to exclude individuals from the study. These criteria were established to ensure sample homogeneity and to avoid potential medical, physiological, and lifestyle-related confounders that could affect vitamin status.

**Table 2:** Baseline Characteristics of Study Participants (*n* = 91).

Characteristic	Total (*n* = 91)	Males (*n* = 27)	Females (*n* = 64)	*p*-Value
**Demographics**				
Age (years), mean ± SD	19.8 ± 1.4	20.1 ± 1.5	19.7 ± 1.3	0.234
Academic year, *n* (%)				
- Year 1–2	51 (56.0)	14 (51.9)	37 (57.8)	
- Year 3–4	30 (33.0)	10 (37.0)	20 (31.3)	
- Year 5–6	10 (11.0)	3 (11.1)	7 (10.9)	
**Anthropometrics**				
BMI (kg/m^2^), mean ± SD	23.2 ± 2.9	24.3 ± 3.1	22.7 ± 2.7	0.018 *
Waist circumference (cm)	76.4 ± 9.8	84.2 ± 8.1	73.1 ± 8.6	<0.001 ***
Body fat (%), mean ± SD	24.8 ± 7.3	18.6 ± 5.2	27.4 ± 6.8	<0.001 ***
**Lifestyle Factors**				
Physical activity (min/week)	142 ± 86	184 ± 92	124 ± 78	0.002 **
Sleep duration (hours/night)	6.4 ± 1.2	6.6 ± 1.1	6.3 ± 1.2	0.268
Stress score (0–10)	7.1 ± 1.6	6.6 ± 1.7	7.3 ± 1.5	0.049 *
Study hours/week	42.3 ± 12.1	39.8 ± 11.4	43.3 ± 12.3	0.213
**Dietary Patterns**				
Fruit/vegetable servings/day	2.9 ± 1.4	3.0 ± 1.5	2.8 ± 1.4	0.547
Fast food meals/week	3.3 ± 2.0	3.7 ± 2.2	3.1 ± 1.9	0.201
Skip breakfast ≥ 3/week, *n* (%)	38 (41.8)	9 (33.3)	29 (45.3)	0.292
Coffee intake (cups/day)	2.1 ± 1.3	2.3 ± 1.4	2.0 ± 1.2	0.314

* *p* < 0.05; ** *p* < 0.01; *** *p* < 0.001. **Legend:** Baseline demographic, anthropometric, lifestyle, and dietary characteristics of the study population stratified by gender. Data are presented as mean ± standard deviation for continuous variables and *n* (%) for categorical variables. *p*-values were calculated using independent *t*-tests for continuous variables and chi-square tests for categorical variables. BMI, body mass index; MET, metabolic equivalent of task.

**Table 3:** Key Water-soluble Vitamin Levels and Deficiency Prevalence.

Vitamin	Mean ± SD	Median (IQR)	Reference Range	Deficiency *n* (%)	Insufficiency *n* (%)	Normal *n* (%)
**Cobalamin** (pg/mL)	485.3 ± 165.0	446.4 (375.2–569.2)	300–900	0 (0.0)	8 (9.0)	81 (91.0) *
**Folate** (ng/mL)	14.1 ± 4.9	13.1 (10.4–16.1)	>5	0 (0.0)	0 (0.0)	89 (100.0) *
**Vitamin C** (µmol/L)	56.7 ± 14.8	55.1 (49.6–62.0)	40–75	2 (2.2)	5 (5.5)	84 (92.3)

* *n* = 89 for cobalamin and folate due to insufficient sample volume in two patients. **Legend:** Serum levels of water-soluble vitamins in the study population (*n* = 91). Data are presented as mean ± standard deviation and median (interquartile range). Deficiency and insufficiency were defined according to established clinical cutoffs: cobalamin deficiency < 200 pg/mL, insufficiency 200–300 pg/mL; folate deficiency < 3 ng/mL, insufficiency 3–5 ng/mL; vitamin C deficiency < 28 µmol/L, insufficiency 28–40 µmol/L. IQR, interquartile range; SD, standard deviation.

**Table 4:** Gender Differences in Vitamin Status.

Vitamin	Males (*n* = 27)	Females (*n* = 64)	*p*-Value	Effect Size (Cohen’s d)
**Cobalamin (pg/mL)**	524.6 ± 178.3	468.7 ± 157.2	0.146	0.33
**Folate (ng/mL)**	13.4 ± 4.7	14.3 ± 5.0	0.431	0.18
**Vitamin C (µmol/L)**	60.2 ± 15.9	55.2 ± 14.1	0.142	0.33
**B12 < 300 pg/mL, *n* (%)**	1 (3.7)	7 (10.9)	0.433 ‡	-
**Vit C < 40 µmol/L, *n* (%)**	0 (0.0)	7 (10.9)	0.096 ‡	-

**Legend:** Comparison of water-soluble vitamin levels between male and female medical students. Data are presented as mean ± standard deviation for continuous variables and *n* (%) for categorical variables. *p*-values were calculated using independent *t*-tests for continuous variables. ‡ Fisher’s exact test was used for categorical variables due to small expected cell frequencies. Cohen’s d effect sizes: 0.2 = small, 0.5 = medium, 0.8 = large. SD, standard deviation.

**Table 5:** Correlations Between Vitamin Levels and Lifestyle Factors.

Variable	Vitamin C	Vitamin B12	Folate
**Dietary Factors**			
Fruit/vegetable intake	0.412 ***	0.156	0.234 *
Fast food frequency	−0.287 **	−0.143	−0.089
Breakfast regularity	0.198 *	0.167	0.156
**Lifestyle Factors**			
Physical activity	0.156	0.098	0.123
Sleep duration	0.167	0.145	0.134
Stress score	−0.241 *	−0.089	−0.098
**Anthropometrics**			
BMI	−0.198 *	−0.067	0.045
Body fat %	−0.234 *	−0.098	−0.056

* *p* < 0.05; ** *p* < 0.01; *** *p* < 0.001; Pearson’s correlation coefficients. **Legend:** Pearson correlation coefficients between serum vitamin levels and lifestyle factors. Fruit/vegetable intake measured as servings per day; fast food frequency as times per week; breakfast regularity as binary variable (regular = 1, irregular = 0); physical activity as MET-minutes per week; sleep duration as hours per night; stress score on 0–10 scale; BMI as kg/m^2^; body fat as percentage. BMI, body mass index; MET, metabolic equivalent of task.

**Table 6:** Multivariate Logistic Regression for Suboptimal Vitamin Levels.

Predictors	Coefficient	Standard Error		*p*-Value	Adjusted Odds Ratio	95% Confidence Interval for Odds Ratio
**Model 1: Vitamin C < 40 µmol/L**						
Fruit/vegetable < 3 servings/day	1.57	0.66	0.018 *	4.8	1.3–17.6	
Stress score ≥ 8	1.16	0.54	0.033 *	3.2	1.1–9.4	
Female gender	1.06	0.58	0.071	2.9	0.9–9.1	
**Model 2: Vitamin B12 < 300 pg/mL**						
Female gender	1.28	0.60		0.032 *	3.6	1.1–11.8
Irregular meals	1.03	0.51		0.045 *	2.8	1.0–7.9
Vegetarian tendency		0.83	0.56	0.138	2.3	0.8–6.7

* *p* < 0.05; Models adjusted for age, academic year, and physical activity. Hosmer–Lemeshow test: Model 1 *p* = 0.58; Model 2 *p* = 0.71. **Legend:** Multivariate logistic regression analysis identifying independent predictors of suboptimal vitamin C status (defined as <40 µmol/L). Variables with *p* < 0.20 in bivariate analysis were included in the initial model, followed by backward stepwise selection with retention threshold *p* < 0.05. The model correctly classified 84.6% of cases. β, regression coefficient; SE, standard error; OR, odds ratio; CI, confidence interval; BMI, body mass index..

**Table 7:** Diagnostic Performance of Screening Tools for Vitamin Deficiency Risk.

Screening Tool	Sensitivity (%)	Specificity (%)	PPV (%)	NPV (%)	AUC (95% CI)
**For Vitamin C Deficiency**					
Combined model	85.7	77.4	28.6	98.1	0.812 (0.721–0.903)
**For B12 Insufficiency**					
Combined model	75.0	73.5	20.0	97.1	0.743 (0.634–0.852)

PPV, positive predictive value; NPV, negative predictive value; AUC, area under the curve; CI, confidence interval. **Legend:** Diagnostic performance characteristics of various screening approaches for identifying students at risk for vitamin deficiencies. Combined models incorporate multiple predictors using logistic regression. PPV, positive predictive value; NPV, negative predictive value; AUC, area under the receiver operating characteristic curve; CI, confidence interval.

**Table 8:** Association Between Vitamin Status and Self-Reported Outcomes.

Outcome	Normal Vitamins	Any Suboptimal	*p*-Value
Fatigue score (0–10)	5.2 ± 1.8	7.4 ± 1.5	<0.001
URI episodes/year	1.8 ± 1.1	3.0 ± 1.4	0.003
Concentration difficulty, *n* (%)	28 (35.0)	9 (75.0)	0.009
Academic performance (self-rated)	7.2 ± 1.3	6.1 ± 1.5	0.012

URI: Upper respiratory infection. **Legend:** Association between suboptimal vitamin status and self-reported clinical and academic outcomes. The “Any Suboptimal” group includes participants with insufficient or deficient levels of either Vitamin C or Vitamin B12. Data are presented as mean ± standard deviation for continuous variables and *n* (%) for categorical variables. *p*-values were calculated using independent *t*-tests for continuous outcomes and chi-square tests for categorical outcomes. URI, Upper respiratory infection.

**Table 9:** A Conceptual Framework for a Risk-Stratified Screening Protocol for Vitamin C and Vitamin B12 Insufficiency in Students.

Tier	Identification of Students	Proposed Action
**Tier 1:** **Universal Prevention**	All students	Promote access to healthy food options; Provide an educational module on nutrition during academic stress.
**Tier 2:** **Targeted Risk Assessment**	Students with key risk factors (poor diet, high stress, female gender, etc.)	Administer risk questionnaire; Perform targeted biochemical screening based on risk profile
**Tier 3:** **Comprehensive Management**	Students with confirmed deficiencies from Tier 2 testing.	Conduct complete micronutrient panel; Provide individualized dietary plan and counseling; Consider supplementation.

**Legend:** A conceptual framework for a tiered approach to nutritional screening in a university student population. This framework is derived from the risk factors identified in this study and is proposed as a model for future validation. It prioritizes low-cost, universal education (Tier 1), followed by targeted risk assessment (Tier 2) to identify students who would most benefit from biochemical testing and comprehensive management (Tier 3).

## Data Availability

The data presented in this study are available on request from the corresponding author. The data are not publicly available due to privacy restrictions, however de-identified data is available from the corresponding author upon reasonable request.
